# HA-FPN: Hierarchical Attention Feature Pyramid Network for Object Detection

**DOI:** 10.3390/s23094508

**Published:** 2023-05-05

**Authors:** Jin Dang, Xiaofen Tang, Shuai Li

**Affiliations:** School of Information Engineering, Ningxia University, Yinchuan 750021, China; 12021131688@stu.nxu.edu.cn (J.D.);

**Keywords:** transformer, feature pyramid networks, object detection, attention modules

## Abstract

The goals of object detection are to accurately detect and locate objects of various sizes in digital images. Multi-scale processing technology can improve the detection accuracy of the detector. Feature pyramid networks (FPNs) have been proven to be effective in extracting multi-scaled features. However, most existing object detection methods recognize objects in isolation, without considering contextual information between objects. Moreover, for the sake of computational efficiency, a significant reduction in the channel dimension may lead to the loss of semantic information. This study explores the utilization of attention mechanisms to augment the representational power and efficiency of features, ultimately improving the accuracy and efficiency of object detection. The study proposed a novel hierarchical attention feature pyramid network (HA-FPN), which comprises two key components: transformer feature pyramid networks (TFPNs) and channel attention modules (CAMs). In TFPNs, multi-scaled convolutional features are embedded as tokens and self-attention is applied to across both the intra- and inter-scales to capture contextual information between the tokens. CAMs are employed to select the channels with rich channel information to alleviate massive channel information losses. By introducing contextual information and attention mechanisms, the HA-FPN significantly improves the accuracy of bounding box detection, leading to more precise identification and localization of target objects. Extensive experiments conducted on the challenging MS COCO dataset demonstrate that the proposed HA-FPN outperforms existing multi-object detection models, while incurring minimal computational overhead.

## 1. Introduction

**Figure 1:**
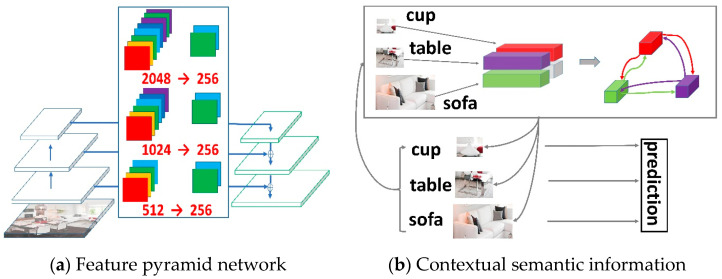
(**a**) This figure shows that other levels of ignored features may provide useful information for the final prediction; reducing many channel dimensions has led to significant losses in channel information. (**b**) Global scene contextual information is transmitted to each level.

At present, it is challenging for object detectors to detect and locate multiple objects at different scales. As each layer of a convolutional neural network (CNN) [1] has a fixed receptive field, Regional CNNs (R-CNNs) encounter specific issues [2]. For instance, there tend to be discrepancies between the fixed receptive fields and the objects in the natural images at different scales. In many current object detectors, pyramid feature representation is used to alleviate those problems [3]. As shown in Figure 1a, top-down architecture was used in this study to produce more semantic feature maps at all scales [4]. Specifically, it integrates low-resolution and semantically strong features with high-resolution and semantically weak features through lateral connections. In recent years, many studies have been conducted to improve the performance of FPNs. To increase the representation of low-level information in deep layers, Path Aggregation Networks (PANs) [5] based on feature pyramid networks (FPNs) have been proposed to add bottom-up pathways. Along with pathway augmentation, Neural Architecture Search FPN (NAS-FPN) [6] was proposed for a more effective fusion of all cross-scale connections. Additionally, augmented FPN (AugFPN) [7] has been proposed in recent research, which utilizes residual feature augmentation to gain ratio-invariant contextual information. However, the aforementioned methods consider each object independently and, therefore, do not take into account the relationships between objects or between objects and their surroundings. As a result, the accuracy of object detection has certain limitations. In this study, it is believed that these approaches neglect the information provided at other scales, and uniformly scaled feature maps representing non-local interactions are insufficient to capture contextual information.

**Figure 2:**
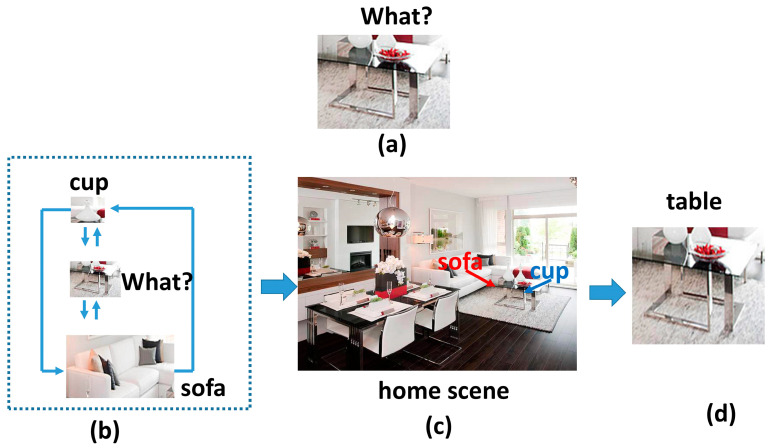
This figure shows an example of the ignored features which could potentially provide important prediction information. When an object (table) is shown independently, it is difficult to recognize. However, with the availability of information concerning the surrounding environment, the table in the illustration can be promptly identified.

Figure 2 provides an illustration of the ignored features that could potentially yield crucial prediction information. In some cases, it is challenging for humans to recognize objects or their respective locations, as depicted in Figure 2a. Nevertheless, as illustrated in Figure 2b, the presence of a cup and a sofa in a home setting aids in the identification of the target object as a table. Coexisting objects provide strong cues for detecting specific objects, as exemplified in Figure 2c, where the points surrounding the cup and sofa tend to identify the table. In Figure 2d, when the surrounding environment information is given, we can then recognize the table easily. Additionally, global scene clues can prevent objects from being wrongly detected in unsuitable surroundings. For instance, a cup is more likely to be located on a table than on a road, and a desk is more likely to be situated in front of a sofa than a car. Therefore, this study posits that contextual information for object detection consists of multiple levels.

Contextual information has been demonstrated to play a vital role in semantic segmentation and object detection processes [8]. For the extraction of context across multiple scales, the Deeplab-v2 [9] pyramid pooling has been implemented in pyramid scene parsing networks to obtain a hierarchical global context, significantly enhancing the semantic segmentation quality of extraction context across multiple scales. Incorporating contextual information can also enhance the final detection and classification results by facilitating the localization of region proposals. Additionally, the use of contextual information in salient object detection (SOD) has been introduced in several recent studies. For example, a novel cross-level attention mechanism in the SOD network was proposed in the cross-level attention and supervision of salient object detection (CLASS) [10] by modeling the channel-wise and position-wise dependency trends between features at different levels. 

**Figure 3:**
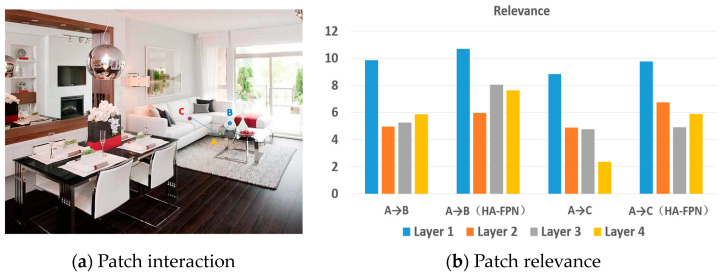
This figure illustrates the analysis of the similarity of attention scores by different layers.

The method of effectively integrating context information exchange at different scales using a transformer is worth studying. The transformer [11] is an architecture that does not use convolutional operators and solely relies on attention mechanisms. The vision transformer is based on learning attentive interaction between distinct patch tokens and has recently received considerable interest in many vision tasks. In addition, the vision transformer (ViT) [12] and data-efficient image transformer (DeiT) [13] methods can partition images into patch embedding sequences, then input them into conventional transformers in image classification challenges. Recently introduced methods make targeted adjustments to ViT which effectively enhance image categorization performance. Additionally, the Cross-attention Multi-scale Vision Transformer (CrossViT) [14] employs a dual-branch transformer to process picture patches of varying sizes, while the Twins [15] approach blends local and global attention techniques to improve feature representation. The results of the above-mentioned studies have shown that transformer-based models outperform other types of networks. In this study, a transformer module was introduced to model multi-scale global scene contexts. As illustrated in Figure 1b, compared to methods based on convolutional neural networks, the proposed transformer can capture long-range dependencies between pixels and global contexts of models. As depicted in Figure 3a, we randomly sampled cups (Patch A in yellow) to analyze patch interactions between the table (Patch B in blue) and the sofa (Patch C in red). Further, we performed analysis of the similarity of attention scores across different layers (Figure 3b), and upon adding the proposed HA-FPN model, attention score similarities of different levels of the table, cup, and sofa significantly improved.

In order to improve computational efficiency, an FPN reduces the channel dimensionality, which results in significant loss of channel information as shown in Figure 1a. The channel dimensions are reduced from 2048, 1024, and 512 to 256. In an attention mechanism, more resources will be invested in the most important feature maps by determining the differences in the importance of each feature map. In squeeze-and-excitation networks (SE-Nets) [16], each channel is assigned a weight to assist the networks to learn important features. More efficient channel attention networks (ECA-Nets) [17] improve the SE-Net blocks by obtaining more accurate attention information via one-dimensional convolution layers for consolidating cross-channel information. Then, with the introduction of selective kernel networks (SK-Nets) [18], the adaptive receptive field sizes of the neurons were achieved through the nonlinear integration of information from multiple kernels. The convolutional block attention module (CBAM) [19] collects spatial and channel attention information by constructing two submodules. Then, it integrates the information, thereby yielding more comprehensive and reliable attention information. Therefore, inspired by the above-mentioned methods, this study introduced a channel attention module that effectively utilized the channels containing rich channel information.

This study proposed a method named HA-FPN. A transformer feature map fusion method was first proposed to combine feature maps of different scales in various layers. It enabled the model to learn global contextual semantic information. Then, an effective yet simple channel attention module was presented for selecting the key channels. This effectively utilized the channels with rich channel information. It also alleviated the problems of massive channel information losses. 

By replacing the FPN with the HA-FPN in a Faster R-CNN, the proposed model achieved a performance improvement of 1.6 and 1 AP, respectively, when using ResNet-50 and ResNet-101 as backbones. Additionally, using ResNet-50 as an initial network, the proposed HA-FPN improved Faster R-CNN [20] by 1.5 AP. In addition to two-stage detectors, with minor modifications, the HA-FPN was also successfully applied to one-stage detectors. The results revealed that RetinaNet [21] was improved by 1.2 AP by replacing the FPN with the HA-FPN. Therefore, the proposed HA-FPN has universality in object detection challenges.

The main contributions of this study can be summarized as follows:

- The proposed TFPN could fully utilize multilevel features in the FPN, which captured global scene information;
- The proposed CAM could successfully invest more computing resources of the neural networks into the most important channels, which alleviated the problem of massive channel information losses;
- This proposed HA-FPN was based on the two contributions mentioned above and was designed to be a simple and generic algorithm that can boost performance detection, while remaining computationally efficient on multiple object detectors.

## 2. Related Work

As can be seen in the literature, the feature pyramid network can be used to detect smaller objects on shallow features and larger objects on deep features to achieve more accurate detection. However, using features of lower or higher levels alone still cannot meet the requirements of high-quality detection. If the global context information can be properly used, the detector can prevent the detection in inappropriate scenarios. In addition, the transformer displayed the ability to capture long-range dependencies between pixels and global contexts of the model.

### 2.1. Feature Pyramid Networks

**Figure 4:**
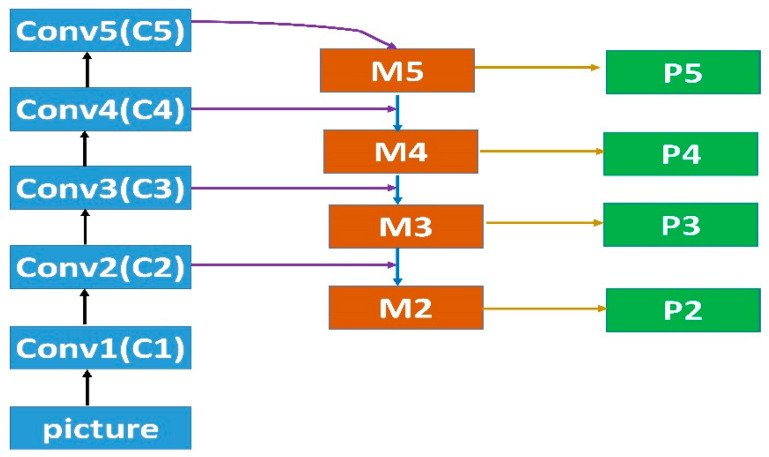
This figure illustrates the feature pyramid network-specific process.

As shown in Figure 4, a feature map of the FPN is first continuously downsampled and a group of feature layers (C1, C2, C3, C4, C5) with high semantic content is obtained. However, less effective information will be obtained regarding the smaller objects on the feature map, and the detection of those smaller objects will decline sharply. The up-sampling process is then re-performed several times to enlarge the feature map to its original size, which will generate feature layers (M2, M3, M4, M5). In that way, the characteristics and information of the smaller targets can be guaranteed to the greatest extent. Finally, the down-sampled features and up-sampled features are fused, and a group of feature layers (P2, P3, P4, P5), with both good spatial information and strong semantic information, is obtained.

### 2.2. Transformer

**Figure 5:**
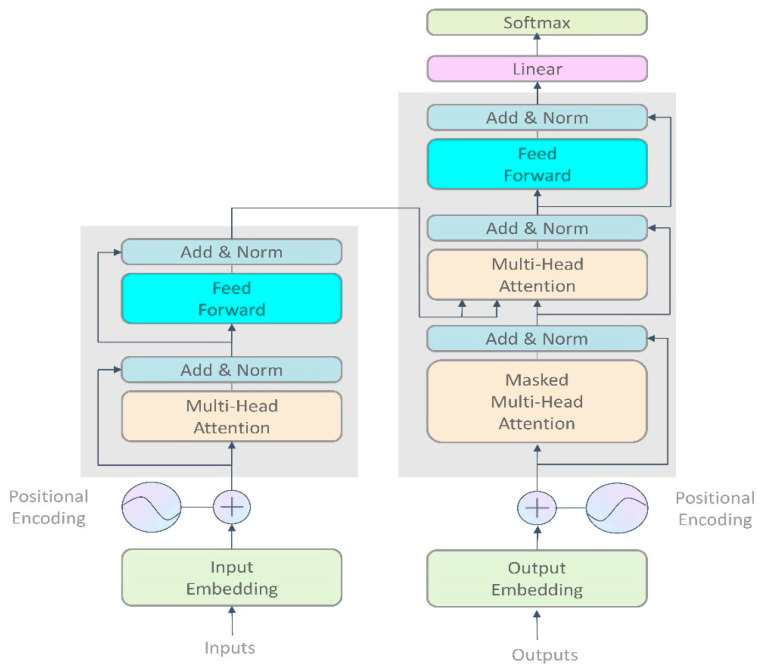
This figure illustrates the diagram of a transformer.

In recent years, transformers have gained significant success in the field of computer science due to their advantages, such as parallelism and the ability to handle long-range dependencies. The general architecture of a transformer is illustrated in Figure 5. However, as transformers do not inherently learn sequence position information, position encoding is typically introduced into input sequences, which enables a model to learn global structural information. Moreover, transformer models use multi-head attention modules to extract more comprehensive features from multiple perspectives and levels. Additionally, residual structures and normalization layers are incorporated between different modules, which effectively prevent the issue of gradient disappearance and accelerate the training process.

### 2.3. Contextual Information

When compared with traditional object detection models, the accuracy of object detection models based on deep learning has been greatly improved in recent years. However, the detection accuracy of those models on the Microsoft common objects in context (MS COCO) dataset [22] remains relatively low. This is due to the presence of objects at different scales and complex backgrounds with serious overlapping between objects in the MS COCO dataset. However, effective fusion of output features from different convolutional layers can obtain relevant contextual information, thereby improving the recognition efficiency of objects of various scales. Moreover, fusing contextual information in the features for classification and regression can effectively alleviate the negative influence of complex backgrounds on accuracy. For instance, when detecting a specific car in an image, objects that typically coexist with the target (e.g., people, roads, or other cars) can provide useful clues for object detection. Therefore, to achieve more accurate results, it is necessary to identify useful contextual information. In addition to the global context of an image, it can also be seen that the regions surrounding a target can provide some useful hints for inferring the content of the target. For example, the surroundings (road) and parts of the target objects (wheels) help detect the objects (cars).

### 2.4. Attention Mechanisms

Attention mechanisms have become increasingly popular in various domains and are often used in the form of weights. In computer vision tasks, different regions of an image or frames of a video can have varying degrees of importance, and attention mechanisms have been found to process visual information more flexibly. The underlying principle of attention mechanisms is similar to the selective attention mechanism in humans. When humans observe an object, they tend to ignore irrelevant information and focus only on what is useful for decision making. Attention mechanisms are based on the idea of obtaining information that is critical to the task. Therefore, attention mechanisms are generally used to automatically select features or regions of interest in object detection processes.

## 3. Materials and Methods

**Figure 6:**
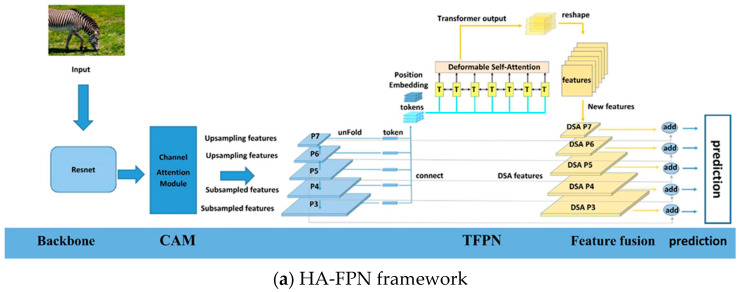

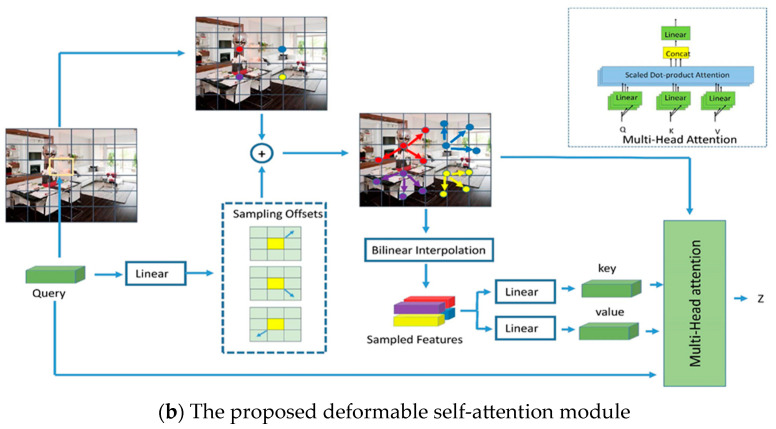
This figure illustrates the pipeline of the HA-FPN-based detector: TFPNs capture global scene contextual information from multi-scaled feature maps, and then this information is transmitted to each level; CAM captures the channels with rich channel information.

The proposed method introduces an HA-FPN. The overall HA-FPN framework is shown in Figure 6a. The proposed HA-FPN consisted of two components: transformer feature pyramid networks (TFPNs) and channel attention modules (CAMs).

### 3.1. Transformer Feature Pyramid Networks

In the overall TFPN framework shown in Figure 6a, the backbone network is ResNet-50 [23], and the feature levels generated by the FPN are [*P3*, *P4*, *P5*, *P6*, *P7*].

**Figure 7:**
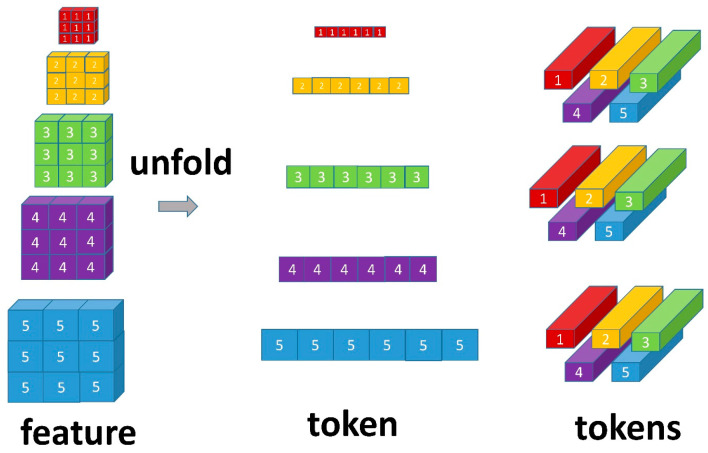
This figure illustrates the unfolding operation.

As shown in Figure 7, this study used an unfolding operation for a 2D feature map, in which $x^{i}\in R^{H_{i}\times W_{i}\times C_{i}}(i=3,4,5,6,7)$ denotes the feature maps for each feature level; *H* and *W* are the height and width of the feature map, respectively; and *C* denotes the number of channels. This study first reshaped the feature map ${x^{i}\in R}^{H_{i}\times W_{i}\times C_{i}}$ into a sequence of flattened 1D tokens $T_{i}\in R^{p_{i}\times C_{i}}$, *P = H × W*. Then, all the feature maps were flattened at the feature level to tokens $T_{i}\in R^{p_{i}\times C_{i}}(i=3,4,5,6,7)$. Finally, the position information is very important for the transformer, so this study added the $T_{p}$ to retain the positional information.

Next, transformer modules were used to model the multi-scale global scene contexts. As in the original transformer, its attention operation was calculated as follows:

(1) $$Attention(q,k,v)=Softmax\left(\frac{qk^{Τ}}{\sqrt{d}}\right)v$$

In the attention mechanism, a score ($qk^{T}$) is computed for each pair of vectors. According to those scores, the different vectors received different levels of attention. Next, $\sqrt{d}$ enhances gradient stability and leads to more stable model training. The *Softmax* function is applied to calculate the probabilities based on the scores, and the values of the vectors are multiplied by the sum of the probabilities. The vectors with higher probability received additional attention from the subsequent layers.

In the original transformer, the computational complexity was relatively high. However, in deformable attention [24], several key points are identified within each promising region. Then, features around these key points are sampled at an appropriate scale, as illustrated in Figure 6b. To address the convergence issues and high computational complexity, this study assigned only a small fixed number of keys to each query. Therefore, the approach adopted in this study replaced attention with deformable self-attention (*DSA*). The multi-head deformable self-attention (*MDSA*) process can be described as follows:

(2) $$MDSA\left(LN\left(T_{i-1}\right)\right)=Concat\left({DSA}_{1},\ldots ,{DSA}_{h}\right)W_{o}^{l}$$

where LN(·) denotes the layer normalization operator and $W_{o}\in R^{hd_{k}\times C}$ is a parameter of the output linear projection head. Therefore, for the l−th layers, the input to the *DSA* was computed from the input $T_{l-1}$.

The transformer encoder is composed of *L* layers of the multi-head deformable self-attention (*MDSA*) and multilayer perceptron (*MLP*) blocks. Therefore, the output of the l layers could be written as follows:

(3) $$\begin{array}{c} T_{i}^{\prime }=MDSA\left(LN\left(T_{i-1}\right)\right)+T_{i-1}\\ T_{i}=MLP\left(LN\left(T_{i}^{\prime }\right)\right)+T_{i}^{\prime } \end{array}$$

The *MLP* was composed of two layers with Gaussian Error Linear Unit (GELU) nonlinearity. After multi-scale context modeling, the tokens $T_{i}$ were reshaped as an image in the spatial dimension:

(4) $$M=\;\mathrm{Reshape}\left(T_{i}\right)$$

Then, the reshape process reorganized tokens from $T_{i}\in R^{l\times C}$ to $M\in R^{h\times w\times c}$, where l is the length of $T_{i}$; h, w, c are the height, width, and channel, respectively, and l=h×w. The features {*DSA* *P*3, *DSA* *P*4, *DSA* *P*5, *DSA* *P*6, *DSA* *P*7} with contextual semantic information were obtained. Finally, feature fusion was carried out through add(·):

(5) $${\mathrm{feature}}_{i}=P_{i}+DSAP_{i},(i=3,4,5,6,7)$$

### 3.2. Channel Attention Module

**Figure 8:**
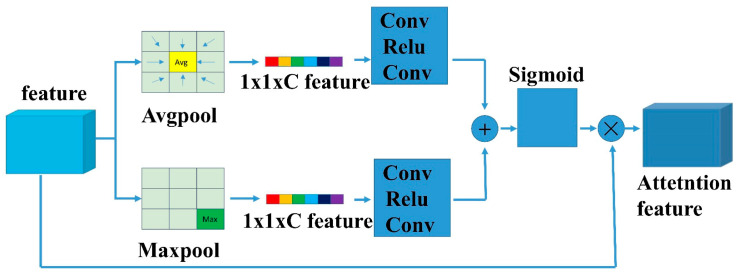
The figure illustrates the CAM.

The overall CAM framework is detailed in Figure 8. The output of one convolution block was represented by $X\in R^{W\times H\times C}$, where W, H, and C are the width, height, and channel dimensions, respectively. To aggregate two kinds of spatial contextual information, this study used average pooling and max pooling methods independently. The average pooling and maximum pooling operations were calculated as follows:

(6) $$\begin{array}{c} g_{\mathrm{Avg}}(X)=\frac{1}{WH}\sum_{i=1,j=1}^{W,H}X_{ij}\\ g_{\max}(X)=\max\left(X_{ij}\right),(i\in W,j\in H) \end{array}$$

where $X_{ij}$ represents the value of the feature map. The average-pooling $g_{Avg}(X)$ and max-pooling $g_{max}(X)$ were applied to the $X_{ij}$ based on its width and height. Then, two feature maps, $F_{Avg}\in R^{l\times l\times C}$ and $F_{max}\in R^{l\times l\times C}$, were obtained. The maps were then fed into a two-layer MLP:

(7) $$f_{\left\{w_{1},w_{2}\right\}}(y)=W_{2}ReLU\left(W_{1}y\right)$$

Neural networks consist of two layers. The first layer ($w_{1}$) had c/r (*r* is the reduction rate) neurons, and a Rectified Linear Unit (ReLU) function was responsible for the activation. The second layer ($w_{2}$) had c neurons. Then, the output features of the MLP were added based on Elementwise and then activated by Sigmoid to generate the final channel attention features as follows:

(8) $$M=\mathrm{sigmoid}\left(f_{\left\{w_{1},w_{2}\right\}}\left(g_{Avg}(X)\right)+f_{\left\{w_{1},w_{2}\right\}}\left(g_{\max}(X)\right)\right)$$

## 4. Experiments

This section presents experimental evaluations of the proposed method on a publicly available dataset and conducts comparative analysis against existing state-of-the-art techniques using multiple evaluation metrics. Our findings indicate that the proposed approach significantly improves the representation power and utilization efficiency of features, leading to enhanced performance of existing detectors. Additionally, an ablation study is conducted to ascertain the contribution of each component of the proposed method. Finally, the experimental results are visualized to understand the regions and features that the neural network focuses on the targets, thereby improving the model’s interpretability and reliability.

### 4.1. Dataset and Evaluation Metrics

In this study, the MS COCO dataset was adopted for all experiments. Due to the intricate nature of the MS COCO dataset, segmentations and object detection pose challenges. To compare the obtained results, all experiments were executed using PyTorch and mm detection [25].

### 4.2. Main Results

Table 1 shows the results using ResNet-50 as the initial network. Mask R-CNN achieved the highest performance by replacing the FPN with the HA-FPN. Specifically, AP, AP_L_, AP_M_, and AP_S_ have achieved 1.6, 2.8, 0.9, and 0.6 promotions when compared to the FPN (ResNet-50), and AP had 1 promotion compared to the FPN (ResNet-101). Furthermore, Faster R-CNN achieved 38.9 AP with ResNet-50 as the initial network, which was 1.5 points higher than the baseline, after replacing the FPN with the HA-FPN. In addition to the two-stage frameworks, this study further extends the HA-FPN to one-stage detectors. The results indicate that it achieves a significant improvement, ranging from 35.9 AP to 37.1 AP, compared to RetinaNet, a typical one-stage detector.

The proposed HA-FPN consistently improved various detectors, as demonstrated in Table 1. Furthermore, the HA-FPN was found to be both robust and generalizable in the conducted experiments. As evident from the AP_S_, AP_M_, and AP_L_ columns, the proposed model achieved an overall detection improvement. In comparison to other state-of-the-art detectors, the HA-FPN obtained competitive outcomes. The experimental outcomes of this study suggested that the proposed HA-FPN was beneficial in all types of detection.

### 4.3. Visualization Results

**Figure 9:**
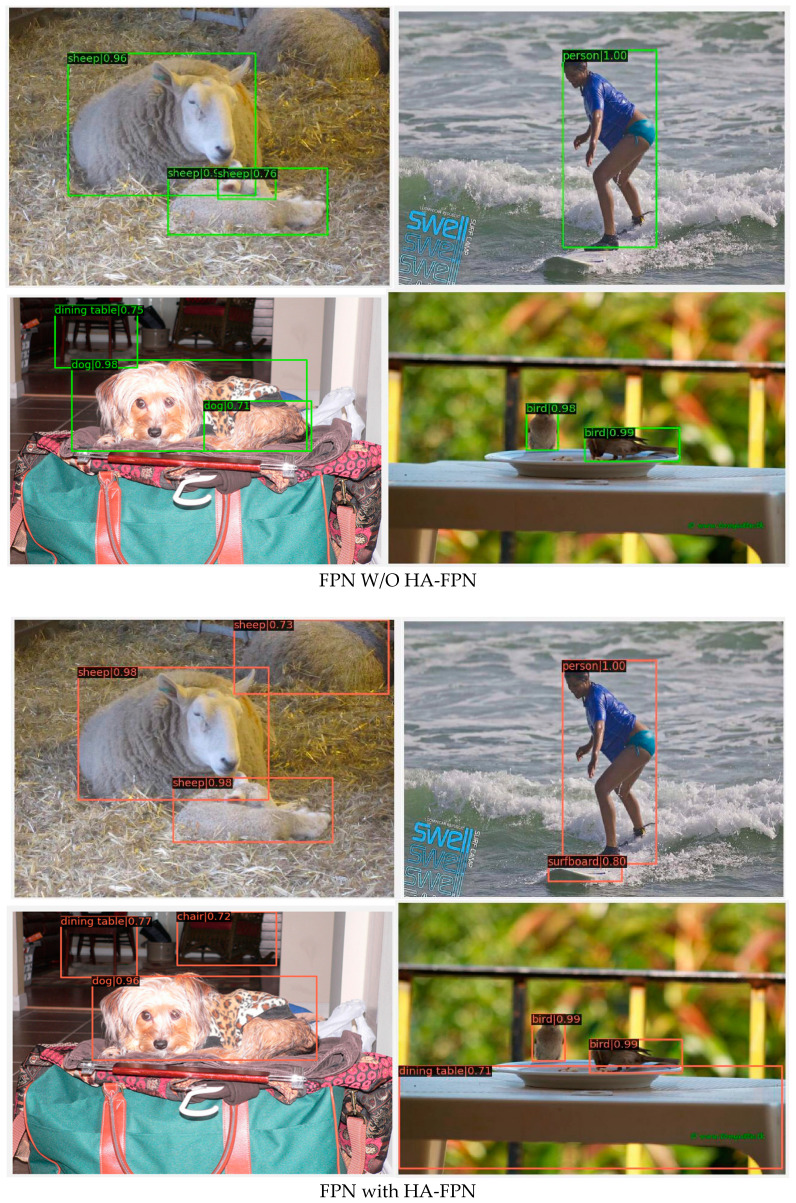
This figure illustrates results of Mask R-CNN with (w) and without (w/o) HA-FPN built upon ResNet-50 on MS COCO test-dev.

**Figure 10:**
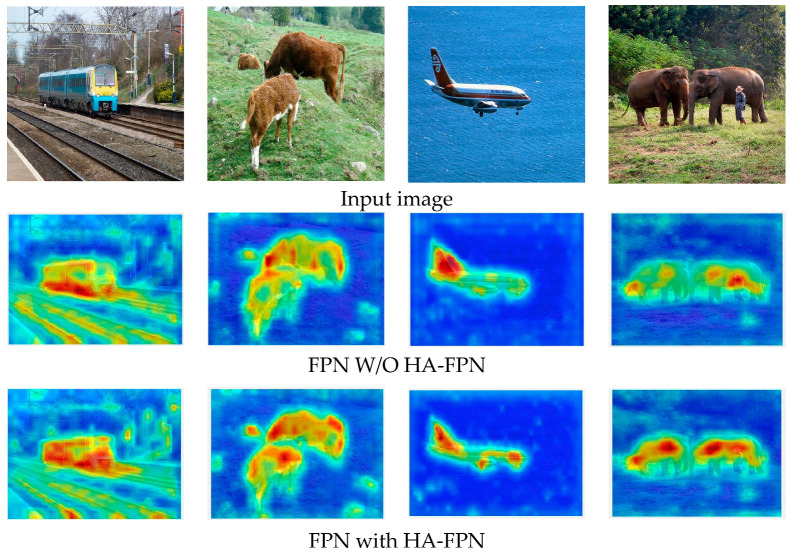
This figure illustrates impacts of HA-FPN via visualizing heat maps on MS COCO test-dev.

We performed detection experiments on the FPN with and without the HA-FPN on the Mask R-CNN network with ResNet-50 as the backbone. The visualization results are depicted in Figure 9. The IoU threshold was set to 0.7. Compared to the typical FPN, the HA-FPN provided satisfactory results, showing better performance in detecting objects of small sizes and those outside the receptive fields. The HA-FPN model was also found to be more effective in multilevel information interaction, because it makes use of information-rich channels. To further study the impacts of HA-FPN, the visualization results of the heat map were examined, as detailed in Figure 10. The results showed that the FPN with the HA-FPN covered the target object regions more effectively than the FPN without the HA-FPN, indicating that the proposed HA-FPN model could achieve enhanced prediction regions.

**Figure 11:**
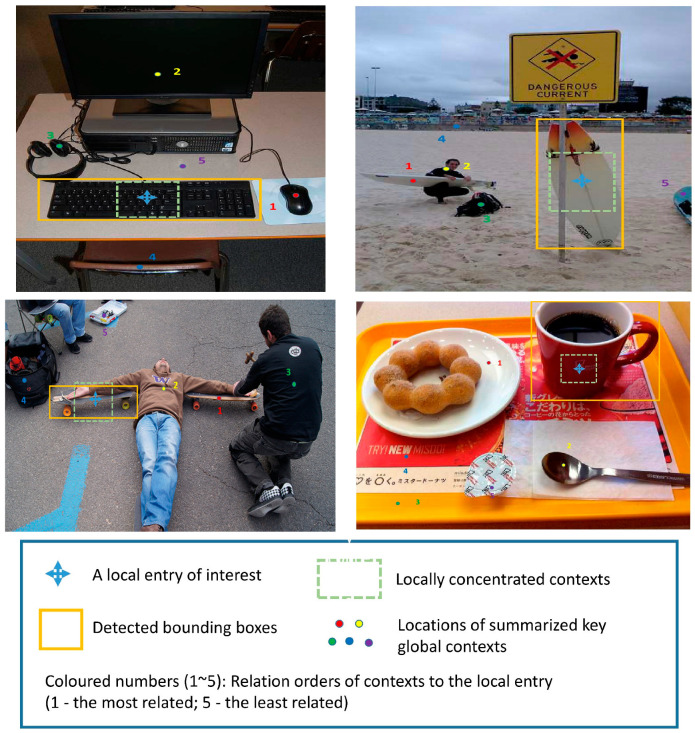
This figure illustrates spatial arrangements of condensed context and its relationship to an entity from a specific local entry.

**Figure 12:**
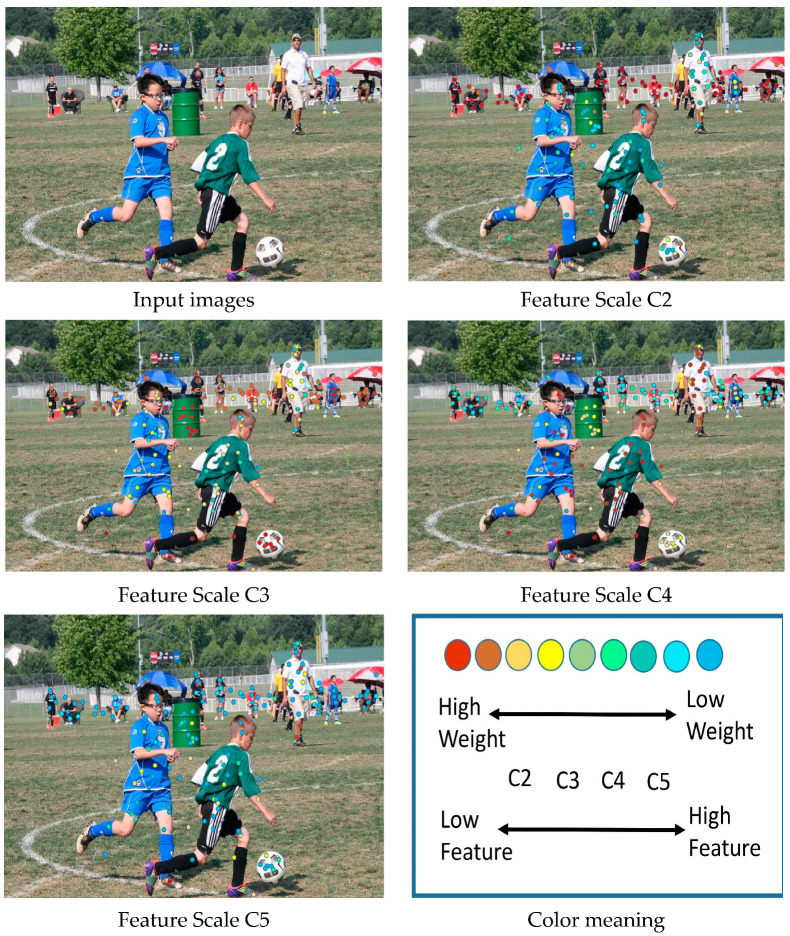
This figure illustrates the weight changes of objects of different scales in different feature layers.

To investigate the relationship between global context and local features, we visualized the correlation between different objects. A visualization of this study’s results is shown in Figure 11. The results achieved by the HA-FPN are shown as visualizations for the deepest-level features. As can be seen in the figure, this study mainly presented the local feature (blue cross). It has locally concentrated contexts (green dotted rectangle) and the global context key points (colored dots). In addition to location, the degrees of correlation between different contextual information and local features are also indicated. In the figure, the relationship order is represented by colored numbers as follows: 1 is the most relevant and 5 is the least relevant. The results showed that the relationships between the global context key points and local features seemed natural. For instance, the HA-FPN model effectively utilized the mouse, computer screen, and computer earphone as the most useful cues for detecting the keyboard, while the chair and the desk were less important, which is consistent with common sense deductions. These findings demonstrate that the HA-FPN can effectively model the relationships between global context and local features, thus enhancing the efficiency of object detection. To investigate the model’s changes in detecting multi-scale objects, we visualized the weight changes of objects at different scales in different levels, as shown in Figure 12. The weight visualizations mainly fall around the objects of interest, and many are semantically rich sampling points. In addition, the HA-FPN can adaptively select appropriate feature scales and generate weights containing rich information.

### 4.4. Ablation Study

A series of ablation experiments were conducted in this section to examine the effects of the individual components of the proposed HA-FPN. The baseline method for all the ablation studies was Faster R-CNN with ResNet-50.

#### 4.4.1. Ablation Studies on the Importance of Each Component

The overall ablation studies are presented in Table 2 to verify the effectiveness of each proposed component. The Faster R-CNN of the ResNet-50 backbone network was gradually augmented with TFPN and CAM. As shown in Table 3, the transformer FPN improved the baseline method by 1.1 AP. This improvement could be attributed to the information interactions across multiple levels of features, allowing the model to learn global contextual information. Additionally, the channel attention module improved the detection performance from 37.4 to 37.7 AP, indicating that the model effectively utilized channels with rich channel information. The HA-FPN achieved 38.9 AP, with a 1.5 AP improvement when two components were incorporated into the baseline method. The aforementioned results suggest that these two components addressed different problems in the FPN and complemented each other.

#### 4.4.2. Ablation Dataset Studies

In the field of vision tasks, convolutional architecture has proven to be highly effective. However, recent experimental results have shown that vision transformers (ViTs) outperform CNNs for image classification due to their use of self-attention layers that provide global features. Nevertheless, ViTs require significant data pretraining before they can be used. In this study, the transformer FPN was developed to combine the strengths of both architectures while avoiding their respective limitations. As shown in Table 3 the transformer FPN remained robust in VOC 2007 [27], demonstrating its potential for use in vision tasks with limited data. Therefore, this study analyzed the reasons behind these results. The analysis revealed that the transformer used one-dimensional sequences as inputs and focused solely on global modeling at all stages, resulting in low-resolution features and a lack of detailed location information. However, the FPN architecture provided highly detailed, high-resolution spatial information, which compensated for the transformer’s shortcomings.

#### 4.4.3. Ablation Studies of the Network Structures of Multi-Scale Feature Map Information Interactions

Table 4 presents a comparison of three different network structures for feature fusion. When using a CNN, nonlocal [28], and transformer structure, the baseline method was improved by 0.4 AP, 0.7 AP, and 1.1 AP, respectively. These experimental results demonstrate that feature fusion across multiple levels can aid in accurate predictions. Furthermore, the transformer achieved the best performance, and it displayed significant advantages in multi-scaled feature fusion. The analysis of this study revealed that traditional CNN-based models were limited in their ability to model long-term dependencies due to the local properties of their convolutional kernels. In contrast, the transformer had global self-attention mechanisms for learning more contextual information among multi-scaled features. Through the self-attention mechanism, the transformer exchanged information between all input pairs, capturing different object types of dependencies and thereby capturing more semantic meaning among the multi-scaled features. The fusion of CNN requires both up-sampling and down-sampling processes, which may result in a considerable loss of information. However, the transformer does not require the same size feature map structure as H*W*C, thereby having the advantage of multi-scaled feature map fusion.

#### 4.4.4. Ablation Studies of Transformer Feature Pyramid Networks

As shown in Table 5, this study compared different feature fusion methods. It was found that the feature fusion method of Add improved the baseline method by 1.1 AP. In contrast, the Concat fusion method led to a decrease in accuracy. Subsequently, a detailed analysis of the differences between the Add and Concat fusion methods was carried out. 

The Concat method splices multiple feature maps along the channel dimension, resulting in an increase in the number of channels. On the other hand, the Add feature fusion method is a pixel overlay with the same number of channels. The Add feature fusion method was much less computationally intensive than Concat. The former increases the amount of information in the feature descriptions while maintaining the dimensions of the image descriptions. However, the amount of information and contextual semantic information in each dimension increased, which was found to be beneficial to the final image classification process. In the contextual semantic information propagation process, the Add feature fusion method was found to achieve a better performance than Concat. For example, the latter had many more channels, thereby giving it many more choices which may effectively learn the relationships between the targets and context.

#### 4.4.5. Ablation Studies of the Channel Attention Module

As shown in Table 6, this study compared the performance differences between the convolutions and the fully connected channels in the CAM. The results showed that both the convolutions and fully connected channels improved the baseline method by 0.4 AP. However, a 5 × 5 convolution degraded the accuracy. The findings of this study indicate that the fully connected (FC) method involves breaking down the feature graph to form a one-dimensional vector, which is then multiplied by a weight vector to obtain an output value. In contrast, the convolution kernel represents the weight, and a 1 × 1 convolution kernel consists of only one weight. The CAM first employs average pooling and maximum pooling for each channel independently. The feature graph will be 1 × 1, and the 1 × 1 convolution output is also a value. Therefore, a 1 × 1 convolution is equivalent to being fully connected. The experimental results showed that a 1 × 1 convolution and a full connection could achieve the same performance in the channel attention module. In this study’s experiments, the size of the convolution kernel was changed to 5, and the model’s performance was observed to decrease. Therefore, ResNet-50 may prefer a smaller kernel size in the HA-FPN.

#### 4.4.6. Ablation Studies of the Spatial Attention

As seen in Table 7, this study compared CAM with CBAM and found that the addition of CAM significantly improved the performance of the model. However, the spatial attention module of CBAM did not contribute to the networks as much as CAM. This study hypothesized that the main reasons were as follows: firstly, the locations of attention extraction and application were not reasonable; secondly, attention was placed on the weights that were attached to features, both enhanced and unenhanced. Thus, when a large number of weights did not achieve the expected enhancement effects, the AP did not improve. 

It can be observed that the accuracy of the model was greatly reduced when only CBAM was added. This study suggests that the following reasons may account for the decrease in accuracy: Firstly, CBAM utilizes convolutional layers to encode local spatial information, which is not enough to capture long-term dependencies in the absence of TFPN. Secondly, the addition of the spatial attention module resulted in broken feature maps, which further affected the model’s accuracy. 

#### 4.4.7. Ablation Studies of the Small Objects with Limited Data

**Figure 13:**
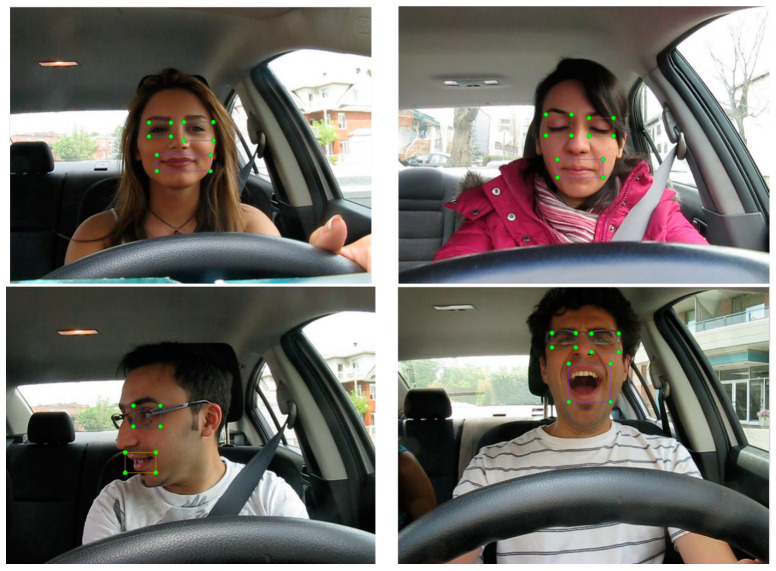
Annotation diagram.

To test the detection performance of the proposed HA-FPN method in practical application scenarios where object sizes are small and data sizes are limited, an image detection dataset of fatigue driving was created. First, the YawDD [29] dataset was used as the video dataset, and videos were converted to image format. The self-made dataset includes 733 images of 26 subjects from the videos, with different facial expressions, with and without wearing glasses, and different genders. The labeling tool LabelImg was used to label the eye and mouth features in the images, with a total of four categories, where open_eye represents open eyes, closed_eye represents closed eyes, and open_mouth and closed_mouth correspond to open and closed mouth, respectively. After labeling, LabelImg generated corresponding XML format annotation files for the images. The labeled images are shown in Figure 13, where the area enclosed by the green dots represents the annotated regions of the eyes and mouth.

The experimental results in Table 8 show that the TFPN has the most significant improvement, with an increase of 3.4 points, and the CAM has an improvement of 2.6 points. This demonstrates the effectiveness of the proposed methods in practical applications, as each method has significantly improved the detection system. Moreover, both the eyes and mouth are small targets, the training dataset contains only slightly over 500 images (compared to the COCO dataset which has over 110,000 images), and the proposed methods have consistently improved the performance of the object detector, especially in scenarios where object sizes are small and data sizes are limited.

## 5. Conclusions

Most of the FPN-based methods suffer from the design defects of channel reduction and underutilization of multi-scale information. Based on those observations, a new feature pyramid network, named HA-FPN, was proposed. The HA-FPN consisted of two main components: a transformer FPN and a channel attention module. Through the transformer FPN, the model learned global contextual semantic information from features that were ignored by other layers. Additionally, the channel attention module effectively utilized channels with rich channel information and alleviated the problem of massive channel information losses. This study demonstrated on the challenging MS COCO dataset that the proposed HA-FPN model provided significant detection improvements. Results from the MS COCO dataset confirmed that the proposed HA-FPN exhibited good generalization for both one- and two-stage detectors. In practical application scenarios, experiments conducted on a self-made dataset showed that the proposed method could significantly improve detector performance, especially for small object detection with limited data.

## Figures and Tables

**Table 1:** This table presents the comparison with the state-of-the-art methods on MS COCO test-dev. The * symbol indicates that this study reimplemented results through mm detection.

Method	Backbone	Schedule	AP	AP_50_	AP_75_	AP_S_	AP_M_	AP_L_	Flops	Params
Baseline:										
RetinaNet * [21]	ResNet-50	1×	35.9	54.9	38.1	20.1	39.7	46.7	250.34	37.74
Faster-RCNN * [20]	ResNet-50	1×	37.4	58.7	40.5	21.7	40.7	48.1	215.82	41.53
Faster-RCNN * [20]	ResNet-101	1×	39.7	60.8	43	24	43.5	51.6	295.7	60.52
Mask R-CNN * [2]	ResNet-50	1×	38	58.9	41.3	22.1	41.7	49.4	268.89	44.17
Mask R-CNN * [2]	ResNet-101	1×	40.4	61.3	44.2	23.4	44.3	53.4	348.77	63.16
State-of-the-art:										
RetinaNet w/AugFPN [7]	ResNet-50	1×	37.5	58.4	40.1	21.3	40.5	47.3		
RetinaNet w/AugFPN [7]	ResNet-50	1×	38.8	61.5	42.0	23.3	42.1	47.7		
Faster-RCNN w/AugFPN [7]	ResNet-101	1×	40.6	63.2	44.0	24.0	44.1	51.0		
Mask R-CNN w/AugFPN [7]	ResNet-50	1×	39.5	61.8	42.9	23.4	42.7	49.1		
Mask R-CNN w/AugFPN [7]	ResNet-101	1×	41.3	63.5	44.9	24.2	44.8	52		
Libra RetinaNet [26]	ResNet-50	1×	37.8	56.9	40.5	21.2	40.9	47.7		
Libra R-CNN [26]	ResNet-50	1×	38.7	59.9	42.0	22.5	41.1	48.7		
Libra R-CNN [26]	ResNet-101	1×	40.3	61.3	43.9	22.9	43.1	51.0		
Ours:										
RetinaNet w/HA-FPN	ResNet-50	1×	37.1	56.3	39.5	22.2	40.9	49.2	256.75	44.04
Faster-RCNN w/HA-FPN	ResNet-50	1×	38.9	60	42.1	22.8	42.5	50.4	241.12	47.83
Faster-RCNN w/HA-FPN	ResNet-101	1×	40.5	61.5	43.9	23	44.4	53.3	321	66.82
Mask R-CNN w/HA-FPN	ResNet-50	1×	39.6	60.5	42.8	22.7	42.6	52.2	294.19	50.47
Mask R-CNN w/HA-FPN	ResNet-101	1×	41.4	62.2	45.1	24.4	44.7	54.9	374.07	69.46

**Table 2:** This table presents the effects of each component: results are reported on MS COCO val2017; TFPN: transformer feature pyramid network; CAM: channel attention module.

Setting	TFPN	CAM	AP	AP_50_	AP_75_	AP_S_	AP_M_	AP_L_
Baseline			37.4	58.7	40.5	21.7	40.7	48.1
	✓		38.5	59	41.6	21.9	41.4	50.1
		✓	37.7	59.2	40.8	21.7	41.7	47.9
	✓	✓	38.9	60	42.1	22.8	42.5	50.4

**Table 3:** This table presents the results of Mask R-CNN with (w) and without (w/o) HA-FPN built upon ResNet-50 on MS COCO test-dev.

Method	Map	Aero	Bike	Bird	Boat	Bottle	Bus	Car	Cat
Faster-RCNN	74.8	76.9	83.2	77.2	62.3	64.6	80.9	84.8	86.5
Faster-RCNN w/HA-FPN	77	82.9	83.3	77.1	64.9	63.2	82.5	86	87.2

**Table 4:** This table presents the ablation studies of the network structures of multi-scaled feature map information interactions on MS COCO val2017. TFPN: transformer feature pyramid network.

Method	Network Structure	AP	AP_50_	AP_75_	AP_S_	AP_M_	AP_L_
Faster-RCNN	None	37.4	58.7	40.5	21.7	40.7	48.1
Faster-RCNN	CNN	37.8 (+0.4)	59.2	40.7	22.2	41.7	48.5
Faster-RCNN	Nonlocal	38.1 (+0.7)	59.5	41.4	22.1	41.8	49
Faster-RCNN w/HA-FPN	Transformer	38.5 (+1.1)	59	41.6	21.9	41.4	50.1

**Table 5:** This table presents the ablation studies of the fusion types of transformer feature pyramid networks on MS COCO val2017. TFPN: transformer feature pyramid networks.

Setting	Fusion Type	AP	AP_50_	AP_75_	AP_S_	AP_M_	AP_L_
Baseline		37.4	58.7	40.5	21.7	40.7	48.1
TFPN	Concat	33.4 (−4)	53.3	35.9	18	38.5	41.9
TFPN	Add	38.5 (+1.1)	59	41.6	21.9	41.4	50.1

**Table 6:** This table presents the ablation studies of multilayered perceptron neural networks of the channel attention module on COCO val2017. TFPN: transformer feature pyramid network; CAM: channel attention module; MLP: multilayered perceptron.

Setting	MLP Type	AP	AP_50_	AP_75_	AP_S_	AP_M_	AP_L_
Baseline		37.4	58.7	40.5	21.7	40.7	48.1
TFPN CAM	1×1 Conv	38.9	60	42.1	22.8	42.5	50.4
TFPN CAM	Fully connected	38.9	59.8	42.3	22	42.3	40.6
TFPN CAM	5×5 Conv	38.5	59.3	41.7	22.2	41.8	50.5

**Table 7:** This table presents the ablation studies of the spatial attention on MS COCO val2017.

Setting	TFPN	CAM	CBAM	AP	AP_50_	AP_75_	AP_S_	AP_M_	AP_L_
Baseline				37.4	58.7	40.5	21.7	40.7	48.1
	✓			38.5	59	41.6	21.9	41.4	50.1
		✓		37.7	59.2	40.8	21.7	41.7	47.9
	✓	✓		38.9	60	42.1	22.8	42.5	50.4
	✓		✓	38.5	58.9	41.6	22	41.2	50.2
			✓	36.9	56	39.1	20.5	40.2	48.9

**Table 8:** Experimental results in practical application scenarios with small targets and limited dataset.

Setting	TFPN	CAM	mAP	Open_Eye	Close_Eye	Close_Mouth	Open_Mouth
Baseline			42.4	17.6	17.7	43.8	90.6
	✓		45.8	30.7	15.2	47.5	89.8
	✓	✓	48.4	21.7	22.2	62	87.8

## Data Availability

The MS COCO datasets and VOC datasets for training and assessment can be found at http://cocodataset.org (accessed on 30 April 2022) and http://host.robots.ox.ac.uk/pascal/VOC/ (accessed on 25 May 2022), respectively.
